# DVL, a Lectin from *Dioclea violacea* Seeds, Disturbs the Proteomic Profile of *Candida krusei,* Leading to Cell Death

**DOI:** 10.3390/antibiotics14121228

**Published:** 2025-12-05

**Authors:** Romério R. S. Silva, Rayara J. P. Carvalho, Maria H. C. Santos, Ana L. E. Santos, Rômulo F. Carneiro, Celso S. Nagano, Pedro F. N. Souza, Claudener S. Teixeira

**Affiliations:** 1 Department of Biochemistry and Molecular Biology, Federal University of Ceará (UFC), Fortaleza 60451-970, CE, Brazil; 2 Medical School, Federal University of Cariri, Barbalha 63180-000, CE, Brazil; 3 Department of Fisheries Engineering, Federal University of Ceará (UFC), Fortaleza 60440-900, CE, Brazil; 4 Laboratory of Bioinformatics Applied to Human Health, Center for Drug Research and Development (NPDM), Federal University of Ceará (UFC), Fortaleza 60355-636, CE, Brazil; 5 National Institute of Science and Technology in Human Pathogenic Fungi (FunVir), Ribeirão Preto 14040-903, SP, Brazil; 6 Visiting Researcher at the Cearense Foundation to Support Scientific and Technological Development, Fortaleza 60325-452, CE, Brazil; 7 Center for Agricultural Sciences and Biodiversity, Federal University of Cariri, Crato 63130-025, CE, Brazil

**Keywords:** oxidative stress, plant lectins, drug resistance, adaptive response

## Abstract

**Background/Objectives** Plant lectins have emerged as potential antifungal molecules, where the carbohydrate recognition domain (CRD) is possibly the main mode of action of these proteins. Previously, we saw that the lectin extracted from the seeds of *Dioclea violacea* (DVL) has anti-candida activity against *Candida krusei* cells by acting to inhibit ergosterol biosynthesis, cell wall deformation, and deregulation of the redox system. **Methods** We have now confirmed this anti-candida activity by proteomic analysis, with the expression of proteins that show us how *C. krusei* cells respond to this treatment. **Results** A total of 395 proteins were identified: 142 proteins exclusively found in untreated *C. krusei* cells and 245 proteins exclusive to DVL-treated cells. Eight proteins were detected in both conditions. Six displayed positive accumulation (fold change > 1.5), one exhibited negative accumulation (fold change < 0.5). We observed the expression of proteins related to cell wall remodeling; alteration of energy metabolism, suggesting a metabolic adaptation to stress; oxidative stress was responded to through the expression of proteins with antioxidant action, in addition to identifying multidrug transport proteins that are often involved in the process of antifungal resistance and sterol transport to the membrane. **Conclusions** Our results show the complexity of adaptive responses of *C. krusei* cells to treatment with DVL, elucidating new mechanisms of resistance and paving the way for the development of more effective and innovative antifungal therapies.

## 1. Introduction

Protein–carbohydrate interactions play a fundamental role in many biological processes and are among the most efficient molecular recognition events in living systems, often triggering complex signaling cascades [1]. Lectins are proteins that bind carbohydrates specifically and reversibly, without altering their chemical structure [2].

Plant lectins have increasingly been recognized as antifungal molecules, with their carbohydrate recognition domains (CRDs) playing a central role in mediating these effects. Prior studies have shown that lectins can exert direct cytotoxicity on fungal cells; for instance, the sunflower mannose-binding lectin Helja selectively damages *Sclerotinia sclerotiorum* and *Fusarium solani*, reducing spore viability after incubation with concentrations as low as 0.05–0.2 μg μL^−1^ [3,4]. In line with this, our group recently demonstrated that the lectin from *Dioclea violacea* seeds (DVL) displays potent antifungal activity against *Candida* species, with MIC_50_ values of 0.6 µM for *C. albicans*, 9.8 µM for *C. krusei*, and 0.6 µM for *C. parapsilosis*. These effects are mediated through multiple mechanisms involving its CRD, including inhibition of ergosterol biosynthesis, alterations in cell wall architecture, and disruption of redox homeostasis [3,4]. Together, these findings highlight lectins as emerging antifungal candidates with well-defined molecular targets.

Understanding such mechanisms is particularly important in the context of rising antifungal resistance. The indiscriminate and repeated use of antifungal agents remains a major driver of resistance, emphasizing the need to elucidate microbial responses to alternative treatments [5].

*Candida krusei* exemplifies this challenge: it is considered a multidrug-resistant pathogen not only because of its high intrinsic resistance to fluconazole (97%), but also due to reduced susceptibility to other antifungal classes [6,7,8]. Reports of increased MICs to polyenes such as amphotericin B, often associated with alterations in ergosterol biosynthesis, and documented cases of echinocandin resistance driven by *FKS1* hotspot mutations reinforce its MDR profile. This combination of resistance mechanisms poses a significant threat to immunocompromised patients and underscores the urgent need for alternative therapeutic strategies in the context of limited antifungal drug development [6,7,8].

Building on our previous findings, the present study deepens the investigation of DVL antifungal activity against *C. krusei* by applying a proteomic approach to characterize the molecular responses triggered by DVL exposure. This analysis provides a broader mechanistic perspective on how this lectin interferes with essential pathways in this multidrug-resistant pathogen.

## 2. Materials and Methods

### 2.1. Biological Material

Seeds of *D. violacea* were collected from plants in Vargem Grande city, Maranhão, Brazil. Seed collection was registered in the National System for the Management of Genetic Heritage and Associated Traditional Knowledge, the SISGEN (Sistema Nacional de Gestão do Patrimônio Genético e Conhecimento Tradicional Associado, ID: AF8E1DD). Regarding microorganisms, the yeasts *C. albicans* (ATCC 10231) were obtained by the Laboratory of Plant Toxins in the Department of Biochemistry and Molecular Biology of the Federal University of Ceará (UFC).

### 2.2. Purification of Lectin from D. violacea Seeds

Lectin was extracted following the protocol of Silva et al. [4]. Briefly, the seeds from *D. violacea* were ground to a fine powder in a coffee mill, and the soluble proteins were extracted at 25 °C by continuous stirring with 50 mL of 0.15 M NaCl (pH 7.0) and 5 g of seed powder for 4 h, followed by centrifugation at 10,000× *g* at 4 °C for 20 min. The use of 0.15 M NaCl as the extraction buffer is standard for Diocleinae lectins, as this ionic strength maintains protein solubility and preserves their native conformation without introducing anions that could interfere with the carbohydrate-recognition domain (CRD).

Protein purification was carried out by the affinity chromatography protocol, using a Sephadex-G75 column (Sigma, Saint Louis, MO, USA) (2 × 10 cm). The fraction containing DVL was then freeze-dried, and purification was judged by SDS-PAGE [9].

### 2.3. Antifungal Assay and Protein Extraction

The antifungal activity of DVL against *C. krusei* was assessed in Sabouraud broth using flat-bottom 96-well microtiter plates according to the Clinical and Laboratory Standards Institute (CLSI) guidelines. An aliquot (50 µL) of yeast cell suspension (0.5–2.5 × 10^6^ CFU mL^−1^) was mixed with 50 µL of DVL at final concentrations ranging from 40 to 0.3 µM, prepared in 0.15 M NaCl (pH 7.0). This NaCl solution served as a neutral vehicle that maintains DVL stability throughout the assay. Plates were incubated for 24 h at 37 °C, and cell growth was measured at 600 nm using a microplate reader (Epoch, BioTek Instruments Inc., Winooski, VT, USA), following CLSI recommendations for turbidity-based fungal growth assessment. Each experiment was performed in triplicate, with three biological replicates. Itraconazole (1000 µg mL^−1^) and 0.15 M NaCl were used as positive and negative controls, respectively. The minimal inhibitory concentration (MIC_50_) was defined as the lowest DVL concentration (µM) that inhibited 50% of yeast growth.

Following the antifungal assay, treated and control cells were immediately processed for protein extraction according to Branco et al. [10]. Samples were washed three times with 50 mM sodium acetate buffer (pH 5.2) and centrifuged at 12,000× *g* for 15 min at 4 °C. Pellets were resuspended in 200 µL of extraction buffer and frozen at −20 °C for 24 h. After thawing, samples were sonicated for 30 min to disrupt the cell wall and plasma membrane. Lysates were centrifuged again, and the supernatant was collected. Protein concentration was determined using the Bradford assay [11], with bovine serum albumin (BSA) as a standard. The resulting protein extracts were used for proteomic analysis.

### 2.4. Gel-Free Proteomic Analysis by LC/MS Mass Spectrometry Analysis

After extraction, a 10 mM DTT (ditiotreitol) solution was added to the samples and incubated for 1 h at 37 °C to reduce the proteins. Iodoacetamide was then added to a final concentration of 15 mM and incubated for a further 30 min in the dark to alkylate the reduced proteins. The proteins were digested with gold trypsin (Promega, Madison, WI, USA) at a final concentration of 1:20 (*w*/*w*), as described by the manufacturer. Trypsin digestion was carried out for 16 h at 37 °C. Finally, the samples were dried in a rapid vacuum (Eppendorf, Hamburg, Germany) for 3 h, resuspended in 0.1% formic acid, and analyzed by nano-HPLC (high-performance liquid chromatography) coupled to an ESI-QUAD-TOF (electrospray ionization quadrupole time-of-flight) mass spectrometer (Waters, Milford, MA, USA).

### 2.5. Protein Identification

Protein identification was carried out according to Branco et al. [10]. The tandem mass spectra were exported as .pkl files and loaded into the MASCOT MS/MS ion search from MATRIX SCIENCE (https://www.matrixscience.com/cgi/search_form.pl?FORMVER=2&SEARCH=MIS, accessed on 10 November 2023) against UP2311_S_cerevisiae (protein database), UP219602_F_oxysporum (protein database), and SwissProt databases (protein database). The search was carried out using the following parameters: fixed modifications in Carbamidomethyl (C), variable modifications in Oxidation (O); the peptide charge was set to 2+, 3+, and 4+; and the instrument was set to ESI-QUAD-TOF. Only proteins with at least two peptides for identification and a false discovery rate of 1% were considered identified.

The identified proteins were searched in UNIPROT and separated into 3 sets: (1) unique to the control for those identified only in the control samples, (2) unique to the cells treated with the DVL lectin for those identified only in the treated samples, and (3) DVL × Control overlapping proteins.

Proteins with a fold change value ≥1.5 (*p* < 0.05, Tukey’s test) were accumulated upwards (increased abundance), and proteins with a fold change value ≤0.5 (*p* < 0.05, Tukey’s test) accumulated downwards (decreased abundance) and were considered for comparisons. Proteins with a fold change value between 0.5 and 1.5 were considered unchanged. The corresponding FASTA file was downloaded for each protein as described by Branco et al. [10]. The blast2go program (https://www.blast2go.com/, accessed on 30 November 2023) was used to categorize the proteins blocked by Gene Ontology (GO) annotation according to molecular function, biological activity, and subcellular location.

### 2.6. Statistical Analysis

All the assays for proteomic analysis and proteomic analysis were performed individually three times, and the values were expressed as the mean ± standard error. The data were submitted to ANOVA software followed by the Tukey test. GraphPad Prism 5.01. version (GraphPad Software Company, San Diego, CA, USA) was used to perform all graphics, with a significance of *p* < 0.05.

## 3. Results and Discussion

### 3.1. Overview

DVL is a lectin isolated and purified from the seeds of *D. violacea*, which has binding specificity to D-glucose and D-mannose [12]. Our recent study indicates that DVL exerts anticandidal activity through multiple mechanisms, with efficacy comparable to that of conventional antifungals. Among the mechanisms, the inhibition of ergosterol biosynthesis and the induction of damage to the membrane and cell wall of fungi stand out. Furthermore, DVL promotes the overproduction of reactive oxygen species (ROS), contributing to its antifungal activity [4].

Deepening the understanding of the mechanisms underlying the anticandidal activity of the DVL lectin, the present study employs advanced proteomic analysis techniques to investigate changes in the protein profile of *C. krusei* cells treated with DVL. Proteomic analysis is a technique that allows a more comprehensive analysis of proteins expressed in a cell or tissue, in which we can understand the role of matrix proteins in pathogenesis, in addition to identifying potential targets for the development of new therapies against *Candida* [13,14].

Another important point is that proteomic analysis is essential to investigating crucial virulence factors during biofilm formation and understanding the steps involved in the acquisition of resistance to azoles [15,16]. This technique also reveals key proteins involved in the response to oxidative stress as potential targets for the development of new antifungals [17].

**Figure 1:**
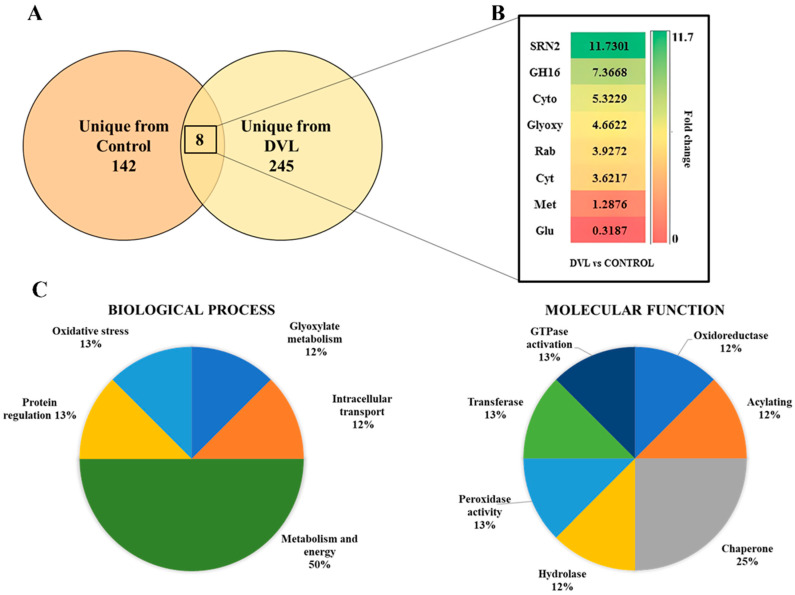
Differentially accumulated proteins in *C. krusei* cells treated with the lectin DVL. In (**A**), the Venn diagram highlights the differential distribution of proteins in single treated and untreated cells from each group and the overlapping protein found in both groups with differential accumulation. (**B**) Proteins are classified according to biological processes. (**C**) Proteins are classified according to molecular function.

A proteomic approach has been carried out to reveal *C. krusei* biofilm cell wall proteins in different concentrations of H_2_O_2_ that protect cells from oxidative stress [18]. Other studies reveal the importance of using proteomics in understanding *Candida* resistance to conventional antibiotics [15,19]. To date, no studies have reported the use of proteomic approaches to examine the response of *C. krusei* to plant lectins. In the present study, LC-ESI-MS/MS analysis was conducted to characterize the protein profiles under different experimental conditions. A total of 395 proteins were identified: 142 in untreated *C. krusei* cells (Table S1), 245 in DVL-treated cells (Table S2), and 8 detected in both conditions (Table 1; Figure 1B). Among these eight shared proteins, six displayed positive accumulation (fold change > 1.5; *p* > 0.05, Tukey test), one exhibited negative accumulation (fold change < 0.5; *p* > 0.05, Tukey test), and one showed no detectable change in abundance (Figure 1A).

Gene Ontology analysis of shared proteins between DVL-treated cells and *C. krusei* control cells revealed the presence of 5 groups related to biological processes (Figure 1C) and 7 groups related to molecular functions (Figure 1C). The largest representation in biological processes was the Metabolism and Energy group, with 50% of the total. In molecular functions, the Chaperone group was the most representative, totaling 25%.

The predominance of proteins related to metabolism suggests a significant influence of the lectin in this process, as was previously observed in a proteomic study involving fluconazole-resistant Candida, where the predominance of metabolic proteins was also highlighted [20]. The hypothesis that the altered expression of these proteins may be associated with antifungal resistance is consistent with our understanding of the role of metabolism in the adaptation of microorganisms to environmental stresses, including treatment with antifungal agents.

### 3.2. Proteins Related to Metabolism and Energy

In this group, we found the protein GH16 domain-containing protein with a positive overlap (Table S1). GH16, also known as glycoside hydrolase family 16, is a poly-specific family of β-glycanases involved in the degradation or remodeling of cell wall polysaccharides [21]. The cell wall, a structure composed of mannoproteins linked to a polysaccharide network, is constantly being remodeled, either during processes such as cell division or due to adaptation to stress factors, which explains the expression of the GH16 protein in both treatments [22]. In stressful situations, such as under the action of antifungal drugs, fungi tend to remodel their cell wall quickly and thus adapt to the composition of a new wall [23,24,25].

Our results indicate that, in *C. krusei* cells, DVL functions as a stressor, exerting direct pressure on the cell wall [4], the up-regulation of GH16 in these cells suggests an adaptive response by *Candida*, possibly involving intensified synthesis and remodeling of cell wall polymers, to cope with antifungal stress and increase its ability to survive and adapt, and that GH16 also plays a role in cell wall biogenesis [26].

In this same group, the Glutamine amidotransferase type-2 domain-containing protein was identified in both treatments with negative regulation (Table S1). Glutamine amidotransferases (GATs) catalyze the hydrolysis of glutamine and transfer the ammonia generated into various metabolites. It is also responsible for catalyzing the first step of the hexosamine pathway, which is essential for the biosynthesis of cell wall precursors. The function of this protein extends to catalyzing the formation of glucosamine 6-phosphate, which is essential for the synthesis of chitin, an essential component of the fungal cell wall [27,28,29]. Therefore, we propose that the negative regulation of the GAT protein indicates a reduction in the production of these precursors, which directly affects the integrity and function of the cell wall. This allows us to understand that the regulation of this protein plays an important role in the homeostasis of cell wall precursors, ensuring the balanced synthesis of chitin, which is important for the structure and protection of fungal cells.

An interesting finding within this group of proteins was that the expression of proteins unique to each treatment reflects important metabolic adaptations; for example, we identified the exclusive presence of the Hexokinase-1 protein in control cells (Table S1). This protein plays a central role in the glycolytic pathway, catalyzing the conversion of glucose into glucose-6-phosphate [30]. This activity not only contributes to energy generation but is also involved in cell survival and virulence mechanisms [31]. The exclusive presence of this protein in control cells suggests that glucose is abundant in this situation, and *C. krusei* cells focus on using this metabolic pathway to sustain growth and reproduction, which does not seem to occur in treated cells, given the absence of this protein.

On the other hand, when *C. krusei* cells were exposed to DVL, we observed a marked metabolic shift, evidenced by the exclusive expression of isocitrate lyase. This enzyme is a key component of the glyoxylate cycle, enabling fungal cells to utilize complex carbon sources, maintain energy production, and generate intermediates essential for gluconeogenesis [32,33]. All this metabolic alteration is fundamental for the survival of these cells in adverse environments, indicating a cellular response to treatment with DVL that can somehow limit the availability of simpler nutrients, such as glucose.

### 3.3. Proteins Related to Cell Wall Organization

**Figure 2:**
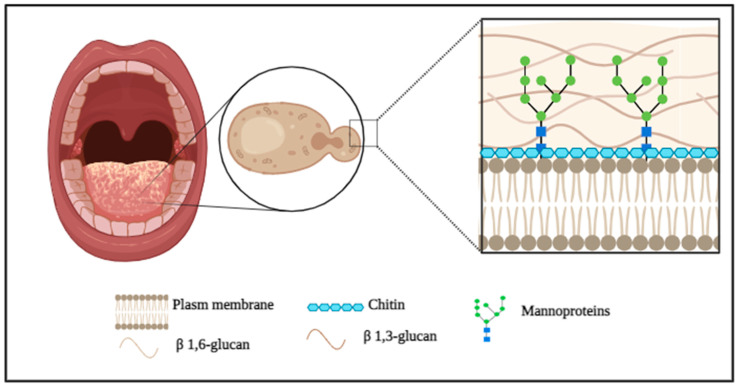
Structural organization and composition of the *C. krusei* cell wall.

The cell wall is an important defense structure in *Candida*, it has high flexibility and adaptive capacity, being able to remodel itself in response to environmental stress or the action of antifungals and the proteins linked to the cell wall play a role in the maintenance and integrity of this wall, in addition, act in virulence processes and are important in the elimination of nutrients [23,34]. Its composition is mainly formed by β-1,3- and β-1,6-glucans (glucose polymers), chitin (a polymer of N-acetylglucosamine), and mannoproteins (Figure 2) [23].

The lectin DVL has been shown to act directly on the cell wall of *C. krusei*, inducing membrane and wall damage that leads to marked structural deformations [4]. In response to these alterations, *C. krusei* cells exposed to DVL exclusively expressed two proteins linked to the biosynthesis and remodeling of cell-wall components (Table S2).

One of the proteins is 1,3-beta-glucanosyltransferase gel4, which is responsible for the synthesis of the cell wall in fungi. It acts by modifying the polysaccharide β-1,3-glucan, the main component of the cell wall in *C. krusei* [35,36]. The exclusive presence of this protein suggests an adaptive response of the cells to the damage caused by the DVL, which is an attempt by these cells to reinforce or remodel the cell wall damaged by the treatment.

Another protein exclusively detected was β-mannosyltransferase 8, an enzyme involved in the synthesis of cell-wall mannans. Mannosyltransferases contribute to the formation of these complex carbohydrate polysaccharides, which are essential for the rigidity and structural integrity of the fungal cell wall [37]. DVL, a lectin with a specific affinity for glucose/mannose, acts by damaging the cell wall [4]. We believe that the exclusive expression of the Beta-mannosyltransferase 8 protein in the treated cells is an adaptive response by the organism to the challenge imposed by treatment with DVL. The exclusive expression of this protein can be interpreted as a compensatory mechanism, increasing the biosynthesis of mannans to strengthen or repair the damaged cell wall. This process suggests an intelligent adaptive mechanism of *C. krusei* cells to resist the damage caused by interaction with the lectin.

### 3.4. Stress and Defense Response Proteins

Within this group, we find the mitochondrial protein Cytochrome c peroxidase, which is up-regulated (Table 1). Under normal conditions, fungal cells tend to generate Reactive Oxygen Species (ROS) as part of their metabolism to act in growth, development, and pathogenicity, but when they are exposed to stressful situations, such as the action of antifungal drugs, these cells tend to overproduce these ROS, making them toxic [38,39]. It is in this sense that antioxidant enzymes come into play to reduce this toxicity. DVL increases the activity of superoxide dismutase (SOD) while reducing the activities of catalase (CAT) and ascorbate peroxidase (APX), ultimately leading to the accumulation of hydrogen peroxide (H_2_O_2_) [4].

Cytochrome c peroxidase acts as an important antioxidant enzyme for the regulation of cellular redox homeostasis by neutralizing ROS, such as H_2_O_2_, which is a potent inducer of ROS [40,41,42]. The positive overlap of this protein in cells treated with DVL suggests a response to oxidative stress, where the increase in this protein is a survival strategy.

Supporting this finding that the expression of antioxidant proteins is essential to combat the ROS generated as a result of the stress caused by DVL, the exclusive presence of glutathione S-transferase omega-like 3 was detected in C. krusei cells exposed to DVL (Table S2). Glutathione transferases are commonly known for their detoxifying role in fungal cells, acting to protect these cells from oxidative stress [43,44].

### 3.5. Transmembrane Transport Proteins

In this group, multidrug transporter proteins were identified exclusively in cells treated with the lectin (Table S2), which are the ABC-type transporter and MFS domain-containing protein. *Candida* species generally use ABC-type and MFS-type multidrug transporter proteins as a mechanism of resistance to antifungal drugs and to facilitate cell survival, thus resisting cell death [45]. These two types of transporters, from different families, are considered the most relevant contributors to multidrug resistance (MDR) in fungi [46].

**Figure 3:**
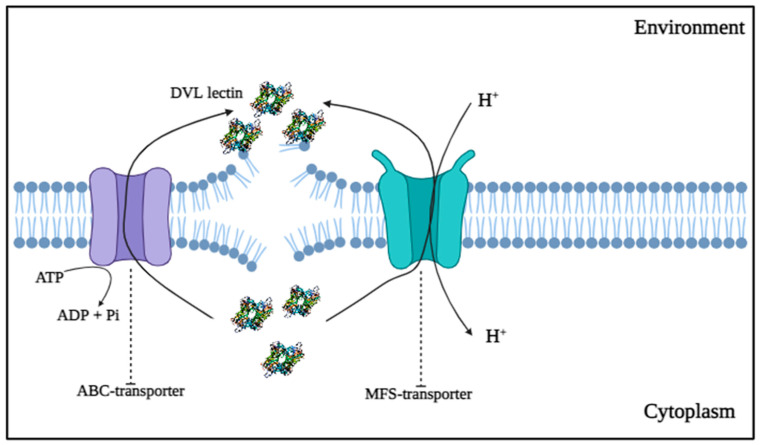
Mechanism of action of the multidrug transporter proteins ABC and MEFS in response to the action of the lectin DVL in *C. krusei*. The ABC multidrug transporter protein (**left**) carries out drug efflux through ATP hydrolysis, while the MFS multidrug transporter protein (**right**) performs efflux through proton translocation.

MFS proteins are considered the largest superfamily of multidrug transporters, and the mechanism by which they mediate hyper-resistance in fungi is through DNA amplification and gain-of-function mutations in transcription factors [47], while ABC proteins contribute to MDR by excluding substrates and showing variable transcriptional response towards antifungal drugs [48]. The translocation of xenobiotics (Figure 3) takes place by coupling to ATP hydrolysis in ABC proteins, and in MFS transporters, this translocation is carried out by means of a proton motive force [49].

We believe that *C. krusei* cells respond to the stress caused by the DVL lectin by expressing these proteins, to export the DVL out of the cell and thus increase their resistance and survival. Another finding in this study is the expression of the MFS domain-containing protein in the control cells (Table S1). This transporter seems to play a basal physiological role, whereas the ABC-type transporter represents an additional defense mechanism activated to counteract the effects of DVL. ABC-type transporters are frequently detected in *Candida* isolates [48,50].

It is worth mentioning that another function attributed to ABC transporters is the maintenance of sterol homeostasis in the plasma membrane. When yeast cells are under some stress, they tend to alter the ergosterol content of the plasma membrane, and one of the ways of replacing ergosterol is through these proteins, acting in the process of remodeling this membrane [51,52]. ABC transporters act not only in the uptake of ergosterol by the plasma membrane, but also in the redistribution of sterols within the membranes [53,54].

The GDP-mannose transporter protein, found exclusively in the treated cells (Table S2), reinforces our previous findings about the importance of cell wall mannan biosynthesis. The GDP-mannose transporter is essential for mannosylation of mannoproteins, glycosylation of mannans that make up the fungal cell wall and can also act as a potential target for antifungal drugs as it plays an important role in mannan biosynthesis [55,56]. The exclusive presence of this protein suggests that *C. krusei* is actively using this biosynthetic pathway to increase the production of mannans and consequently strengthen the cell wall, by virtue of repairing the damage caused by the DVL lectin, which has an affinity for the mannose present in the *C. krusei* cell wall.

We have seen that DVL acts by disturbing the homeostasis of the *C. krusei* redox system, since it increases the activity of the SOD enzyme and decreases the activity of the CAT and APX enzymes, causing an accumulation of H_2_O_2_ [4]. In the cell, this accumulation is reduced precisely by the action of APX, which uses ascorbate (ASC) as an electron donor for H_2_O_2_, reducing it to water and thus balancing the ROS content in these cells. In the absence of APX, the cell seeks alternatives for this H_2_O_2_ reduction through transmembrane transport proteins, such as the protein identified exclusively in the treated cells (Table S2): Cytochrome b561 domain-containing protein (CYB561) [57,58].

CYB561 is a transmembrane protein that acts in the recycling of ASC, and actively acts in antioxidant defense, in this study we suggest that the expression of this protein is an adaptive response of *C. krusei* cells to oxidative stress caused by DVL lectin, whereas CYB561 uses ASC as an electron donor to catalyze the reduction of H_2_O_2_, alternatively by the reduction of APX [58,59,60].

An interesting finding within this group was the expression of the Oxysterol-binding protein homolog 1, only in the treated cells (Table S2). The main function of this protein is to carry out the intracellular transport of sterols, which are essential components of the cell membrane. Ergosterol is the main sterol in fungi and, among its various roles, susceptibility to stress and treatment with antifungals are the most interesting for the production of new drugs [61,62,63].

In our previous work, we saw that DVL acts by inhibiting ergosterol biosynthesis in *C. krusei* cells, interfering with lipid homeostasis and intracellular lipid transport, as well as making fungal membranes more susceptible, now the exclusive expression of the protein, Oxysterol-binding protein homolog 1, suggests an adaptive response of these cells to reward the reduction of ergosterol and consequently adjust lipid metabolism, maintaining the integrity of the cell membrane.

### 3.6. Proteins Related to Lipid Metabolism

Also, as a cellular response to lipid disturbances caused by treatment with the lectin DVL, *C. krusei* cells express the protein Sterol 3-beta-glucosyltransferase. Sterols in Candida are an essential component of membranes, contributing to their fluidity and integrity, and are important for determining the susceptibility of these cells to stress and antifungal treatments [61]. Sterol 3-beta-glucosyltransferase acts in the modification of these sterols and is involved in cell structure, membrane plasticity, anti-stress, heat shock, and signal transduction [64]. The expression of this protein reinforces the hypothesis of an adaptive mechanism in these cells as a result of treatment with DVL lectin, mainly in response to the reduction in ergosterol biosynthesis caused by the treatment.

Given the great importance of the fungal cell wall for intracellular protection, we now discuss, from a lipid point of view, the importance of the fungal plasma membrane, which contains the enzymes and proteins responsible for cell wall biosynthesis, including some lipids, including phospholipids [65]. The importance of the plasma membrane for the survival of fungi in the face of stressors is well known.

Under the effect of treatment with DVL lectin, *C. krusei* cells show a reduction in ergosterol biosynthesis, which can compromise the plasma membrane. In addition to the above, another adaptive response that these cells show to compensate for this reduction is the expression of the Phosphatidylserine decarboxylase proenzyme 3 protein, which is responsible for the biosynthesis of phospholipids and is essential for fungal growth [66]. This protein converts phosphatidylserine into phosphatidylethanolamine, which is one of the main phospholipids found in biological membranes [67]. Increased production of phospholipids via expression of Phosphatidylserine decarboxylase proenzyme 3 has the effect of helping to stabilize the cell membrane and compensate for ergosterol deficiency.

### 3.7. DNA Repair Proteins

In this group, several proteins relevant to fungal survival and proliferation were detected in both treated cells (Table S2) and control cells (Table S1). In the treated cells specifically, the following proteins were identified: proliferating cell nuclear antigen, DNA polymerase lambda, DNA ligase, and DNA topoisomerase 2.

Proliferating cell nuclear antigen (PCNA) is a protein with a central role in cell replication and is involved in several protein factors in response to DNA damage, as well as being a therapeutic target for several diseases [68,69], an important finding is that PCNA accelerates the rate of nucleotide incorporation, carried out by DNA polymerase in yeast cells and helps in the DNA repair process carried out by DNA ligase, that is, the three proteins act concomitantly in the DNA repair process caused by DVL lectin in *C. krusei* cells [70,71]. DNA polymerase and DNA ligase were other proteins expressed in *C. krusei* cells treated with DVL, which perform DNA repair through different pathways [72].

Another protein found exclusively in the treated cells (Table S2) is DNA topoisomerase 2, which is a protein that plays an important role in cell growth and DNA repair, as well as acting on genome integrity [73,74]. The joint expression of these proteins is a response of the cells to treatment with DVL, where both play a significant role in ensuring the replication and repair of DNA damaged by the treatment. This adaptive response allows the treated cells to survive and proliferate even under the stress of treatment with the lectin.

### 3.8. Oxidoreductase-Related Proteins

Within this group, we found the protein: Cytochrome P450 monooxygenase, exclusively in the control cells (Table S1), indicating a possible inhibition of this protein in the treated cells. Commonly, in fungi, P450 is related to primary and secondary metabolic processes, acting in detoxification processes against pollutants and in the degradation of xenobiotic compounds [75,76].

Another important role of Cytochrome P450 monooxygenase is its participation in ergosterol synthesis, virulence processes, and the production of secondary metabolites in fungi [77]. Its absence in cells treated with DVL lectin suggests an interruption of these biosynthetic and detoxification processes and reinforces our findings by directly affecting the integrity of the cell membrane and the ability of *C. krusei* to adapt to treatment.

Lectin-based antifungals represent a promising therapeutic strategy because they exploit a unique mechanism of action. By inducing change in the cell proteomics leading to change in pathways important to cell’s life lectin emerges as a potential antifungal molecule. By doing that, the clinical relevance of lectins relies on two major advantages: (1) a reduced likelihood of cross-resistance with current azole, polyene, or echinocandin therapies, and (2) potentially lower toxicity to human cells. Moreover, lectins may be particularly valuable against multidrug-resistant species. However, clinical translation will require careful optimization to mitigate potential immunogenicity and ensure stability, bioavailability, and safety in vivo.

## 4. Conclusions

The results of this study reveal significant insights into how *C. krusei* cells respond to treatment with the lectin DVL, highlighting the complexity of the cellular mechanisms involved in adapting to stress. Our proteomic analysis revealed the identification of key proteins involved in various biological processes, ranging from energy metabolism, cell wall synthesis, transport proteins, to modification of lipid metabolism, providing a comprehensive view of the strategies adopted by the fungus to face the challenge imposed by DVL. Broadening our view of the mechanisms of resistance of *C. krusei* results in paving the way for more effective and innovative antifungal therapies.

## Figures and Tables

**Table 1:** Overlapping proteins identified in control and treated cells of *C. krusei* by LC-ESI-MS/MS analysis.

Protein	ID (Uniprot)	Organism Reference	Cellular Compartment	Fold-Change DVL vs. NaCl 0.15 M
**Glyoxylate metabolism**				
Glyoxylate reductase 1	P53839	*Saccharomyces cerevisiae* (strain ATCC 204508/S288c)	Cytoplasm	4.662
**Intracellular transport**				
Protein SRN2	Q99176	*Saccharomyces cerevisiae* (strain ATCC 204508/S288c)	Endosome	11.730
**Metabolism and energy**				
Methylmalonate-semialdehyde dehydrogenase (CoA acylating)	A0A2H3I2J3	*Fusarium oxysporum* f. sp. *radicis-cucumerinum*	Cytoplasm	1.288
Glutamine amidotransferase type-2 domain-containing protein	A0A2H3HPJ2	*Fusarium oxysporum* f. sp. *radicis-cucumerinum*	Cytoplasm	0.319
Cytochrome b mRNA-processing protein 4	A0A2H3H0U5	*Fusarium oxysporum* f. sp. *radicis-cucumerinum*	Mitochondrial inner membrane	3.622
GH16 domain-containing protein	A0A2H3HG97	*Fusarium oxysporum* f. sp. *radicis-cucumerinum*	Cell wall	7.367
**Protein regulation**				
Rab-GAP TBC domain-containing protein	A0A2H3HUM8	*Fusarium oxysporum* f. sp. *radicis-cucumerinum*	Cell Membrane	3.927
**Oxidative stress**				
Cytochrome c peroxidase, mitochondrial	Q4WPF8	*Aspergillus fumigatus* (strain ATCC MYA-4609/CBS 101355/FGSC A1100/Af293)	Mitochondrial matrix	5.323

## Data Availability

The original contributions presented in this study are included in the article/Supplementary Materials. Further inquiries can be directed at the corresponding authors.

## Supplementary Materials

The following supporting information can be downloaded at: https://www.mdpi.com/article/10.3390/antibiotics14121228/s1, Table S1: Unique proteins identified in the control-NaCl group by ESI-LC-MS/MS. Table S2: Unique proteins identified in the DVL lectin-treated group by ESI-LC-MS/MS.
