# Supplementary material for: DVL, a Lectin from Dioclea violacea Seeds, Disturbs the Proteomic Profile of Candida krusei, Leading to Cell Death
Source: Antibiotics (Basel). 2025 Dec 5;14(12):1228. doi: 10.3390/antibiotics14121228 (PMC12730034; doi:10.3390/antibiotics14121228)
Supplement: Supplementary file 1 [file antibiotics-14-01228-s001.zip › antibiotics-3963990-supplementary.pdf]

Supplementary Table S1 - Unique proteins identified in the control-NaCl group by ESI-LC-MS/MS

Protein Name	ID Uniprot	Organism Reference	Cellular Compartmet
**Carbohydrate metabolism**			
2-deoxyglucose-6-phosphate phosphatase 1	P38774	*Saccharomyces cerevisiae* (strain ATCC 204508 / S288c)	Cytoplasm
UTP--glucose-1-phosphate uridylyltransferase	P32861	*Saccharomyces cerevisiae* (strain ATCC 204508 / S288c)	Cytoplasm
Glycogen debranching enzyme	Q06625	*Saccharomyces cerevisiae* (strain ATCC 204508 / S288c)	Cytoplasm
Peptide-N(4)-(N-acetyl-beta-glucosaminyl)asparagine amidase	Q02890	*Saccharomyces cerevisiae* (strain ATCC 204508 / S288c)	Cytoplasm
Alpha-galactosidase	A0A2H3HTU1	*Fusarium oxysporum* f. sp. *radicis-cucumerinum*	Cytoplasm
GH43_C2 domain-containing protein	A0A2H3GNT7	*Fusarium oxysporum* f. sp. *radicis-cucumerinum*	Cytoplasm
Probable exo-1,4-beta-xylosidase xlnD	Q0CMH8	*Aspergillus terreus* (strain NIH 2624 / FGSC A1156)	Cytoplasm
**Intracellular Protein Transport**			
Vacuolar protein sorting-associated protein 38	Q05919	*Saccharomyces cerevisiae* (strain ATCC 204508 / S288c)	Endosome
ADP-ribosylation factor GTPase-activating protein GCS1	P35197	*Saccharomyces cerevisiae* (strain ATCC 204508 / S288c)	Cytosol

Protein SBE22	P38814	*Saccharomyces cerevisiae* (strain ATCC 204508 / S288c)	Cytoplasm
Sorting nexin-3	Q08826	*Saccharomyces cerevisiae* (strain ATCC 204508 / S288c)	Cytosol
VWFA domain-containing protein	A0A2H3HJB5	*Fusarium oxysporum* f. sp. *radicis-cucumerinum*	Cytosol
Vacuolar protein-sorting-associated protein 46	P69771	*Saccharomyces cerevisiae* (strain ATCC 204508 / S288c)	Cytoplasm
Protein transport protein SEC24	A5DPC0	*Meyerozyma guilliermondii* (strain ATCC 6260 / CBS 566 / DSM 6381 / JCM 1539 / NBRC 10279 / NRRL Y-324)	Golgi membrane
Myosin-1	A5DKH0	*Meyerozyma guilliermondii* (strain ATCC 6260 / CBS 566 / DSM 6381 / JCM 1539 / NBRC 10279 / NRRL Y-324)	Cytoplasm
**Metabolism and synthesis of amino acids**			
Threonine synthase	P16120	*Saccharomyces cerevisiae* (strain ATCC 204508 / S288c)	Cytoplasm
Homocitrate dehydratase, mitochondrial	P39533	*Saccharomyces cerevisiae* (strain ATCC 204508 / S288c)	Cytosol
PUA domain-containing protein	A0A2H3H509	*Fusarium oxysporum* f. sp. *radicis-cucumerinum*	Cytoplasm
Ornithine transcarbamylase	A0A2H3HT67	*Fusarium oxysporum* f. sp. *radicis-cucumerinum*	Unknown
Probable inactive reductase easA	A2TBU0	*Epichloe festucae* var. *lolii*	Cytoplasm
Bifunctional lycopene cyclase/phytoene synthase	A2QM49	*Aspergillus niger* (strain ATCC MYA-4892 / CBS 513.88 / FGSC A1513)	Membrane
Non-reducing polyketide synthase SAT8	A0A084B9Z6	*Stachybotrys chartarum* (strain CBS 109288 / IBT 7711)	Membrane

Nonribosomal peptide synthetase TES	A0A144KPJ6	*Alternaria alternata*	Membrane
Protein EFR3	Q4P3U5	*Ustilago maydis* (strain 521 / FGSC 9021)	Cytoplasm
2-oxoglutarate-dependent dioxygenase AFUA_1G01000	Q4WKX0	*Aspergillus fumigatus* (strain ATCC MYA-4609 / CBS 101355 / FGSC A1100 / Af293)	Cytoplasm
**Intracellular regulation**			
Intron-encoded DNA endonuclease aI3	P03877	*Saccharomyces cerevisiae* (strain ATCC 204508 / S288c)	Mitochondria
Target of rapamycin complex 2 subunit TSC11	P40061	*Saccharomyces cerevisiae* (strain ATCC 204508 / S288c)	Cytosol
Kinesin-like protein KIP3	P53086	*Saccharomyces cerevisiae* (strain ATCC 204508 / S288c)	Cytoplasmic Microtubule
Transposon Ty1-ML2 Gag-Pol polyprotein	Q03434	*Saccharomyces cerevisiae* (strain ATCC 204508 / S288c)	Cytoplasm
PH domain-containing protein	A0A2H3HLJ8	*Fusarium oxysporum* f. sp. *radicis-cucumerinum*	Cytoplasm
Autophagy-related protein 9	A0A2H3HQJ5	*Fusarium oxysporum* f. sp. *radicis-cucumerinum*	Cytoplasm
Protein kinase domain-containing protein	A0A2H3HM17	*Fusarium oxysporum* f. sp. *radicis-cucumerinum*	Cytoplasm
Guanine nucleotide-binding protein alpha-1 subunit	GPA1_USTMA	*Ustilago maydis* (strain 521 / FGSC 9021)	Cytoplasm

Protein EFR3	P0CN37	*Cryptococcus neoformans* var. *neoformans serotype* D (strain B-3501A)	Membrane
Serine/threonine-protein kinase ste20	STE20_EMENI	*Emericella nidulans* (strain FGSC A4 / ATCC 38163 / CBS 112.46 / NRRL 194 / M139)	Cytoplasm
Probable aspartic-type endopeptidase OPSB	C5FRQ0	*Arthroderma otae* (strain ATCC MYA-4605 / CBS 113480)	Plasm membrane
Peroxisomal membrane protein PEX16	P78980	*Yarrowia lipolytica* (strain CLIB 122 / E 150)	Membrane
**Sporulation**			
Meiotic activator RIM4	P38741	*Saccharomyces cerevisiae* (strain ATCC 204508 / S288c)	Cytoplasm
C2H2 type master regulator of conidiophore development brlA	Q2UQZ5	*Aspergillus oryzae* (strain ATCC 42149 / RIB 40)	Nucleus
Striatin Pro11	Q70M86	*Sordaria macrospora* (strain ATCC MYA-333 / DSM 997 / K(L3346) / K-hell)	Membrane
**Transcription regulation**			
Transcription factor tau 91 kDa subunit	Q06339	*Saccharomyces cerevisiae* (strain ATCC 204508 / S288c)	Nucleus
EKC/KEOPS complex subunit BUD32	P53323	*Saccharomyces cerevisiae* (strain ATCC 204508 / S288c)	Cytoplasm
General transcriptional corepressor CYC8	P14922	*Saccharomyces cerevisiae* (strain ATCC 204508 / S288c)	Nucleus

Histone H2B.2	P02294	*Saccharomyces cerevisiae* (strain ATCC 204508 / S288c)	Nucleosome
Inositol polyphosphate multikinase	P07250	*Saccharomyces cerevisiae* (strain ATCC 204508 / S288c)	Cytoplasm
Zinc finger protein CRM3	Q12145	*Saccharomyces cerevisiae* (strain ATCC 204508 / S288c)	Nucleus
Mediator of RNA polymerase II transcription subunit 1	A0A2H3H7A0	*Fusarium oxysporum* f. sp. *radicis-cucumerinum*	Nucleus
FAR1 domain-containing protein	A0A2H3G5G3	*Fusarium oxysporum* f. sp. *radicis-cucumerinum*	Nucleus
PDCD2_C domain-containing protein	A0A2H3HMF5	*Fusarium oxysporum* f. sp. *radicis-cucumerinum*	Cytoplasm
Fungal_trans domain-containing protein	A0A2H3GJR2	*Fusarium oxysporum* f. sp. *radicis-cucumerinum*	Nucleus
Monodictyphenone cluster transcriptional coactivator mdpA	C8VQ72	*Emericella nidulans* (strain FGSC A4 / ATCC 38163 / CBS 112.46 / NRRL 194 / M139)	Nucleus
Decapping nuclease RAI1	Q6CPU0	*Kluyveromyces lactis* (strain ATCC 8585 / CBS 2359 / DSM 70799 / NBRC 1267 / NRRL Y-1140 / WM37)	Cytoplasm
Pheromone-processing carboxypeptidase KEX1	A8NYP0	*Coprinopsis cinerea* (strain Okayama-7 / 130 / ATCC MYA-4618 / FGSC 9003)	Membrane
Spore development regulator RYP2	B2CQK0	*Ajellomyces capsulatus*	Nucleus
pH-response transcription factor pacC/RIM101	Q9HFB3	*Aspergillus oryzae* (strain ATCC 42149 / RIB 40)	Cytoplasm
**Metabolism and energy**			
Hexokinase-1	P04806	*Saccharomyces cerevisiae* (strain ATCC 204508 / S288c)	Cytoplasm

Dihydrolipoyl dehydrogenase	A0A2H3HMS2	*Fusarium oxysporum* f. sp. *radicis-cucumerinum*	Cytoplasm
**Protein biosynthesis**			
60S ribosomal protein L6-A	Q02326	*Saccharomyces cerevisiae* (strain ATCC 204508 / S288c)	Cytosol
37S ribosomal protein S5, mitochondrial	P33759	*Saccharomyces cerevisiae* (strain ATCC 204508 / S288c)	Mitochondrial Inner Membrane
Ribosomal lysine N-methyltransferase 3	P38222	*Saccharomyces cerevisiae* (strain ATCC 204508 / S288c)	Cytosol
Elongation factor 3B	P53978	*Saccharomyces cerevisiae* (strain ATCC 204508 / S288c)	Ribosome
26S proteasome regulatory subunit RPN3	P40016	*Saccharomyces cerevisiae* (strain ATCC 204508 / S288c)	Cytoplasm
54S ribosomal protein IMG2, mitochondrial	P25642	*Saccharomyces cerevisiae* (strain ATCC 204508 / S288c)	Mitochondrial Inner Membrane
Methionine aminopeptidase	A0A2H3HTU0	*Fusarium oxysporum* f. sp. *radicis-cucumerinum*	Cytoplasm
Eukaryotic translation initiation factor 3 subunit C	Q5AX75	*Emericella nidulans* (strain FGSC A4 / ATCC 38163 / CBS 112.46 / NRRL 194 / M139)	Cytoplasm
Mitochondrial inner membrane i-AAA protease supercomplex subunit MGR3	Q04472	*Saccharomyces cerevisiae* (strain ATCC 204508 / S288c)	Mitochondria
Large ribosomal subunit protein eL6A	Q02326	*Saccharomyces cerevisiae* (strain ATCC 204508 / S288c)	Cytosol

Eukaryotic translation initiation factor 3 subunit G	Q6FRZ6	*Candida glabrata* (strain ATCC 2001 / BCRC 20586 / JCM 3761 / NBRC 0622 / NRRL Y-65 / CBS 138)	Cytosol
Probable tyrosine--tRNA ligase, cytoplasmic	Q8SRV7	*Encephalitozoon cuniculi* (strain GB-M1)	Cytoplasm
Eukaryotic translation initiation factor 3 subunit E	Q0CNR3	*Aspergillus terreus* (strain NIH 2624 / FGSC A1156)	Cytoplasm
**Regulation Factor and RNA Processing**			
25S rRNA (cytosine(2278)-C(5))-methyltransferase	P53972	*Saccharomyces cerevisiae* (strain ATCC 204508 / S288c)	Nucleolus
20S-pre-rRNA D-site endonuclease NOB1	Q08444	*Saccharomyces cerevisiae* (strain ATCC 204508 / S288c)	Cytoplasm
Pre-rRNA-processing protein ESF2	P53743	*Saccharomyces cerevisiae* (strain ATCC 204508 / S288c)	Cytoplasm
Pre-mRNA-splicing factor BRR1	Q99177	*Saccharomyces cerevisiae* (strain ATCC 204508 / S288c)	Nucleus
H/ACA ribonucleoprotein complex subunit GAR1	P28007	*Saccharomyces cerevisiae* (strain ATCC 204508 / S288c)	Nucleolus
Poly(A) polymerase PAPa	Q9UW26	*Candida albicans* (strain SC5314 / ATCC MYA-2876)	Cytosol
Mitochondrial group I intron splicing factor CCM1	A7TMW6	*Vanderwaltozyma polyspora* (strain ATCC 22028 / DSM 70294 / BCRC 21397 / CBS 2163 / NBRC 10782 / NRRL Y-8283 / UCD 57-17)	Mitochondria
Pre-mRNA-processing protein 45	Q5AC37	*Candida albicans* (strain SC5314 / ATCC MYA-2876)	Nucleus

ATP-dependent RNA helicase DBP6	A7TFZ9	*Vanderwaltozyma polyspora* (strain ATCC 22028 / DSM 70294 / BCRC 21397 / CBS 2163 / NBRC 10782 / NRRL Y-8283 / UCD 57-17)	Nucleolus
H/ACA ribonucleoprotein complex subunit GAR1	Q6CJ45	*Kluyveromyces lactis* (strain ATCC 8585 / CBS 2359 / DSM 70799 / NBRC 1267 / NRRL Y-1140 / WM37)	Nucleolus
U2 small nuclear ribonucleoprotein A'	Q6C417	*Yarrowia lipolytica* (strain CLIB 122 / E 150)	Nucleolus
ATP-dependent RNA helicase dbp-10	Q7S9J4	*Neurospora crassa* (strain ATCC 24698 / 74-OR23-1A / CBS 708.71 / DSM 1257 / FGSC 987)	Nucleolus
H/ACA ribonucleoprotein complex subunit GAR1	P28007	*Saccharomyces cerevisiae* (strain ATCC 204508 / S288c)	Nucleolus
Ribosome biogenesis protein ENP2	G0S902	*Chaetomium thermophilum* (strain DSM 1495 / CBS 144.50 / IMI 039719)	Nucleolus
tRNA (guanine(37)-N1)-methyltransferase	Q6CA67	*Yarrowia lipolytica* (strain CLIB 122 / E 150)	Cytoplasm
**Cell cicle**			
Protein STU1	P38198	*Saccharomyces cerevisiae* (strain ATCC 204508 / S288c)	Nucleus
Sister chromatid cohesion protein 1	Q12158	*Saccharomyces cerevisiae* (strain ATCC 204508 / S288c)	Chromosome
Importin alpha re-exporter	P33307	*Saccharomyces cerevisiae* (strain ATCC 204508 / S288c)	Cytosol
MAU2 chromatid cohesion factor homolog	P40090	*Saccharomyces cerevisiae* (strain ATCC 204508 / S288c)	Chromatin

DNA replication licensing factor MCM7	P38132	*Saccharomyces cerevisiae* (strain ATCC 204508 / S288c)	Chromosome
GDP-mannose pyrophosphorylase	A0A2H3GSG6	*Fusarium oxysporum* f. sp. *radicis-cucumerinum*	Cytoplasm
Mannose-1-phosphate guanyltransferase	Q4I1Y5	*Gibberella zeae* (strain ATCC MYA-4620 / CBS 123657 / FGSC 9075 / NRRL 31084 / PH-1)	Cytoplasm
Enhancer of polycomb-like protein 1	Q6CEV5	*Yarrowia lipolytica* (strain CLIB 122 / E 150)	Nucleus
N-acetyltransferase ECO1	Q6C668	*Yarrowia lipolytica* (strain CLIB 122 / E 150)	Chromatin
Sister chromatid cohesion protein pds5	Q9HFF5	*Schizosaccharomyces pombe* (strain 972 / ATCC 24843)	Chromatin
**DNA repair**			
Regulator of Ty1 transposition protein 107	P38850	*Saccharomyces cerevisiae* (strain ATCC 204508 / S288c)	Cell periphery
Non-structural maintenance of chromosome element 4	P43124	*Saccharomyces cerevisiae* (strain ATCC 204508 / S288c)	Nucleus
Chromatin modification-related protein EAF7	P53911	*Saccharomyces cerevisiae* (strain ATCC 204508 / S288c)	Nucleus
General transcription and DNA repair factor IIH helicase subunit XPD	A0A2H3GQC0	*Fusarium oxysporum* f. sp. *radicis-cucumerinum*	Nucleus
Flap endonuclease 1	A8NQC2	*Coprinopsis cinerea* (strain Okayama-7 / 130 / ATCC MYA-4618 / FGSC 9003)	Mitochondria

Topoisomerase 1-associated factor 1	Q6CFJ2	*Yarrowia lipolytica* (strain CLIB 122 / E 150)	Nucleus
Structure-specific endonuclease subunit slx4	Q0C9R2	*Aspergillus terreus* (strain NIH 2624 / FGSC A1156)	Nucleus
Serine/threonine-protein kinase TEL1	Q751J3	*Ashbya gossypii* (strain ATCC 10895 / CBS 109.51 / FGSC 9923 / NRRL Y-1056)	Nucleus
**Transmembrane transporters**			
Protein HOL1	P53389	*Saccharomyces cerevisiae* (strain ATCC 204508 / S288c)	Cell periphery
MFS domain-containing protein	A0A2H3HDE2	*Fusarium oxysporum* f. sp. *radicis-cucumerinum*	Membrane
Mitochondrial inner membrane magnesium transporter MRS2	Q6C8H7	*Yarrowia lipolytica* (strain CLIB 122 / E 150)	Membrane
Alternative oxidase, mitochondrial	Q8NKE2	*Cryptococcus neoformans* var. *grubii serotype* A (strain H99 / ATCC 208821 / CBS 10515 / FGSC 9487)	Mitochondrial Inner Membrane
**Stress and Defense Response**			
Heat shock protein STI1	P15705	*Saccharomyces cerevisiae* (strain ATCC 204508 / S288c)	Cytoplasm
HEME_HALOPEROXIDASE domain-containing protein	A0A2H3GIA0	*Fusarium oxysporum* f. sp. *radicis-cucumerinum*	Cytoplasm
NACHT domain-containing protein	A0A2H3GP22	*Fusarium oxysporum* f. sp. *radicis-cucumerinum*	Cytoplasm
Stress response protein NST1	Q5A2K0	*Candida albicans* (strain SC5314 / ATCC MYA-2876)	Membrane
**Nucleotide metabolism**			

Phosphoribosylformylglycinamidine synthase	P38972	*Saccharomyces cerevisiae* (strain ATCC 204508 / S288c)	Cytoplasm
PNP_UDP_1 domain-containing protein	A0A2H3G4J4	*Fusarium oxysporum* f. sp. *radicis-cucumerinum*	Nucleus
**Oxireductase**			
FAD-binding FR-type domain-containing protein	A0A2H3HWW9	*Fusarium oxysporum* f. sp. *radicis-cucumerinum*	Nucleus
FAD_binding_3 domain-containing protein	A0A2H3FN69	*Fusarium oxysporum* f. sp. *radicis-cucumerinum*	Nucleus
PKS_ER domain-containing protein	A0A2H3GLK9	*Fusarium oxysporum* f. sp. *radicis-cucumerinum*	Nucleus
Cytochrome P450 monooxygenase alt1	Q5KTN3	*Alternaria solani*	Membrane
FAD-dependent monooxygenase FVEG_12630	W7N2P6	*Gibberella moniliformis* (strain M3125 / FGSC 7600)	Nucleus
Cytochrome P450 monooxygenase verL	A0A1U9YHZ8	*Clonostachys rogersoniana*	Membrane
NADH-dependent flavin oxidoreductase iccG	A0A482NAR8	*Talaromyces variabilis*	Nucleus
**Gene regulation**			
Origin recognition complex subunit 2	A0A2H3HF08	*Fusarium oxysporum* f. sp. *radicis-cucumerinum*	Nucleus

DNA helicase	A0A2H3I9X6	*Fusarium oxysporum* f. sp. *radicis-cucumerinum*	Nucleus
Zn(2)-C6 fungal-type domain-containing protein	A0A2H3H0A3	*Fusarium oxysporum* f. sp. *radicis-cucumerinum*	Nucleus
**Lipid metabolism**			
Sphingolipid delta(4)-desaturase	Q5AJX2	*Candida albicans* (strain SC5314 / ATCC MYA-2876)	Membrane
Adenylate-forming reductase cicB	A0A1U8QW91	*Emericella nidulans* (strain FGSC A4 / ATCC 38163 / CBS 112.46 / NRRL 194 / M139)	Cytoplasm
Fumagillin dodecapentaenoate synthase af370	Q4WAY3	*Aspergillus fumigatus* (strain ATCC MYA-4609 / CBS 101355 / FGSC A1100 / Af293)	Membrane
Non-reducing polyketide synthase PKS1	F9XMW3	*Zymoseptoria tritici* (strain CBS 115943 / IPO323)	Unknown
NADH-cytochrome b5 reductase 2	Q7SFY2	*Neurospora crassa* (strain ATCC 24698 / 74-OR23-1A / CBS 708.71 / DSM 1257 / FGSC 987)	Membrane
Fatty acid synthase subunit alpha	S5NIA2	*Cochliobolus carbonum*	Membrane
Erythritol-mannosyl-transferase 1	A0A0D1DZV5	*Ustilago maydis* (strain 521 / FGSC 9021)	Membrane
Lysophospholipase NTE1	Q756Z0	*Ashbya gossypii* (strain ATCC 10895 / CBS 109.51 / FGSC 9923 / NRRL Y-1056)	Endoplasmic reticulum
**Cell wall organization**			
Glucan 1,3-beta-glucosidase 3	O74799	*Schizosaccharomyces pombe* (strain 972 / ATCC 24843)	Cytoplasm
Chitin synthase regulatory factor 3	Q09897	*Schizosaccharomyces pombe* (strain 972 / ATCC 24843)	Cytosol

**Unknown**			
UPF0479 membrane protein YER190C-B	P0CX94	*Saccharomyces cerevisiae* (strain ATCC 204508 / S288c)	Membrane
IML2-like protein YKR018C	P36114	*Saccharomyces cerevisiae* (strain ATCC 204508 / S288c)	Cytoplasm
Amidohydro-rel domain-containing protein	A0A2H3GGJ0	*Fusarium oxysporum* f. sp. *radicis-cucumerinum*	Unknown
PAC domain-containing protein	A0A2H3H260	*Fusarium oxysporum* f. sp. *radicis-cucumerinum*	Unknown
Fido domain-containing protein	A0A2H3HWX3	*Fusarium oxysporum* f. sp. *radicis-cucumerinum*	Unknown
Cnn_1N domain-containing protein	A0A2H3GDH4	*Fusarium oxysporum* f. sp. *radicis-cucumerinum*	Cytoplasm
SCA7 domain-containing protein	A0A2H3H0X3	*Fusarium oxysporum* f. sp. *radicis-cucumerinum*	SAGA Complex
Putative UPF0479 protein YBL113W-A	Q8TGK5	*Saccharomyces cerevisiae* (strain ATCC 204508 / S288c)	Membrane

Supplementary Table S2 – Unique proteins identified in the DVL lectin-treated group by ESI-LC-MS/MS

Protein Name	ID Uniprot	Organism Reference	Cellular Compartmet
**Stress and Defense Response**			
Glutathione S-transferase omega-like 3	Q04806	*Saccharomyces cerevisiae* (strain ATCC 204508 / S288c)	Cytoplasm
Putative protein lysine methyltransferase SET5	P38890	*Saccharomyces cerevisiae* (strain ATCC 204508 / S288c)	Cytoplasm
Heat shock protein homolog SSE2	P32590	*Saccharomyces cerevisiae* (strain ATCC 204508 / S288c)	Cytoplasm
Protein HPH2	P39734	*Saccharomyces cerevisiae* (strain ATCC 204508 / S288c)	Endoplasmic reticulum
Heat shock protein SSA2	P10592	*Saccharomyces cerevisiae* (strain ATCC 204508 / S288c)	Cytoplasm
Stress response protein NST1	Q1E1H4	*Coccidioides immitis* (strain RS)	Cytoplasm
3'(2'),5'-bisphosphate nucleotidase	Q5BCG1	*Emericella nidulans* (strain FGSC A4 / ATCC 38163 / CBS 112.46 / NRRL 194 / M139)	Cytoplasm
Cold shock-induced protein TIR2	P33890	*Saccharomyces cerevisiae* (strain ATCC 204508 / S288c)	Cellular periphery
Heat shock protein 60, mitochondrial	Q09864	*Schizosaccharomyces pombe* (strain 972 / ATCC 24843)	Mitochondrial Inner Membrane
**Transmembrane transporters**			
Oxysterol-binding protein homolog 1	P35845	*Saccharomyces cerevisiae* (strain ATCC 204508 / S288c)	Cytosol

Probable glucose transporter HXT5	P38695	*Saccharomyces cerevisiae* (strain ATCC 204508 / S288c)	Plasm membrane
Vacuolar protein sorting-associated protein 13	Q07878	*Saccharomyces cerevisiae* (strain ATCC 204508 / S288c)	Endossome
Membrane-bound O-acyltransferase GUP2	Q08929	*Saccharomyces cerevisiae* (strain ATCC 204508 / S288c)	Endoplasmic reticulum
Polyamine transporter 2	P53283	*Saccharomyces cerevisiae* (strain ATCC 204508 / S288c)	Cellular Periphery
mRNA transport regulator MTR10	Q99189	*Saccharomyces cerevisiae* (strain ATCC 204508 / S288c)	Cytoplasm
ER-derived vesicles protein ERV29	P53337	*Saccharomyces cerevisiae* (strain ATCC 204508 / S288c)	Endoplasmic reticulum
Cytochrome b561 domain-containing protein	A0A2H3HEB1	*Fusarium oxysporum* f. sp. *radicis-cucumerinum*	Membrane
MFS domain-containing protein	A0A2H3GHU5	*Fusarium oxysporum* f. sp. *radicis-cucumerinum*	Membrane
Calcium-transporting ATPase	A0A2H3H5J4	*Fusarium oxysporum* f. sp. *radicis-cucumerinum*	Membrane
Mitochondrial import inner membrane translocase subunit TIM22	A0A2H3HVZ9	*Fusarium oxysporum* f. sp. *radicis-cucumerinum*	Membrane
ADP-ribosylation factor GTPase-activating protein effector protein 2	P40529	*Saccharomyces cerevisiae* (strain ATCC 204508 / S288c)	Cytoplasm
Amino-acid transporter arg-13	Q01356	*Neurospora crassa* (strain ATCC 24698 / 74-OR23-1A / CBS 708.71 / DSM 1257 / FGSC 987)	Mitochondrial Inner Membrane

Golgi to ER traffic protein 1	Q6BYU3	*Debaryomyces hansenii* (strain ATCC 36239 / CBS 767 / BCRC 21394 / JCM 1990 / NBRC 0083 / IGC 2968)	Membrane endoplasmic reticulum
Protein sat1	O60183	*Schizosaccharomyces pombe* (strain 972 / ATCC 24843)	Cytosol
GDP-mannose transporter	Q0UG89	*Phaeosphaeria nodorum* (strain SN15 / ATCC MYA-4574 / FGSC 10173)	Golgi Membrane
ABC-type transporter adrC	A0A1Y0BRF0	*Penicillium roqueforti*	Membrane
**Cell cycle**			
Altered inheritance of mitochondria protein 24, mitochondrial	P47127	*Saccharomyces cerevisiae* (strain ATCC 204508 / S288c)	Mitochondrial Inner Membrane
Protein SFI1	Q12369	*Saccharomyces cerevisiae* (strain ATCC 204508 / S288c)	Cytoplasm
Origin recognition complex subunit 1	P54784	*Saccharomyces cerevisiae* (strain ATCC 204508 / S288c)	Nucleoplasm
Serine/threonine-protein phosphatase PPQ	P32945	*Saccharomyces cerevisiae* (strain ATCC 204508 / S288c)	Cytoplasm
Anaphase-promoting complex subunit CDC27	P38042	*Saccharomyces cerevisiae* (strain ATCC 204508 / S288c)	Cytoplasm
Kinesin-like protein KIP2	P28743	*Saccharomyces cerevisiae* (strain ATCC 204508 / S288c)	Nucleus
Spindle pole body component SPC105	P53148	*Saccharomyces cerevisiae* (strain ATCC 204508 / S288c)	Mitochondria
Chromo domain-containing protein	A0A2H3GQ63	*Fusarium oxysporum* f. sp. *radicis-cucumerinum*	Cytoplasm
Bud site selection protein 27	P43573	*Saccharomyces cerevisiae* (strain ATCC 204508 / S288c)	Cytoplasm

Structural maintenance of chromosomes protein 2	P41003	*Schizosaccharomyces pombe* (strain 972 / ATCC 24843)	Chromatin
Dynein heavy chain, cytoplasmic	Q9C1M7	*Ashbya gossypii* (strain ATCC 10895 / CBS 109.51 / FGSC 9923 / NRRL Y-1056)	Cell cortex
Meiosis-specific APC/C activator protein AMA1	P50082	*Saccharomyces cerevisiae* (strain ATCC 204508 / S288c)	Cytoplasm
Bud site selection protein RAX1	Q08760	*Saccharomyces cerevisiae* (strain ATCC 204508 / S288c)	Plasm membrane
Protein byr4	Q10951	*Schizosaccharomyces pombe* (strain 972 / ATCC 24843)	Cytoplasm
Nitrogen permease regulator 3	C8VSE7	*Emericella nidulans* (strain FGSC A4 / ATCC 38163 / CBS 112.46 / NRRL 194 / M139)	Nucleus
E3 ubiquitin-protein ligase bre1	Q7S304	*Neurospora crassa* (strain ATCC 24698 / 74-OR23-1A / CBS 708.71 / DSM 1257 / FGSC 987)	Nucleus
**Regulation Factor and RNA Processing**			
Nucleolar complex protein 14	Q99207	*Saccharomyces cerevisiae* (strain ATCC 204508 / S288c)	Nucleolus
U3 small nucleolar RNA-associated protein 22	P53254	*Saccharomyces cerevisiae* (strain ATCC 204508 / S288c)	Nucleolus
Ribonucleases P/MRP protein subunit POP1	P41812	*Saccharomyces cerevisiae* (strain ATCC 204508 / S288c)	Cytoplasm
Methylated RNA-binding protein 1	Q06390	*Saccharomyces cerevisiae* (strain ATCC 204508 / S288c)	Cytoplasm
mRNA-capping enzyme subunit beta	O13297	*Saccharomyces cerevisiae* (strain ATCC 204508 / S288c)	Cytoplasm

U3 small nucleolar RNA-associated protein MPP10	P47083	*Saccharomyces cerevisiae* (strain ATCC 204508 / S288c)	Nucleolus
Ribonuclease P protein subunit	A0A2H3GEN4	*Fusarium oxysporum* f. sp. *radicis-cucumerinum*	Nucleus
tRNA-dihydrouridine synthase 3	A0A2H3HP06	*Fusarium oxysporum* f. sp. *radicis-cucumerinum*	Cytoplasm
Protein phosphatase PP2A regulatory subunit B	A0A2H3I3Q4	*Fusarium oxysporum* f. sp. *radicis-cucumerinum*	Nucleus
ATP-dependent RNA helicase MSS116, mitochondrial	Q750Q4	*Ashbya gossypii* (strain ATCC 10895 / CBS 109.51 / FGSC 9923 / NRRL Y-1056)	Mitochondrial matrix
Dicer-like protein 1	A1DE13	*Neosartorya fischeri* (strain ATCC 1020 / DSM 3700 / CBS 544.65 / FGSC A1164 / JCM 1740 / NRRL 181 / WB 181)	Nucleus
GTPase-activating protein BEM2	Q9HF75	*Ashbya gossypii* (strain ATCC 10895 / CBS 109.51 / FGSC 9923 / NRRL Y-1056)	Cytoplasm
Dicer-like protein 1	Q0UI93	*Phaeosphaeria nodorum* (strain SN15 / ATCC MYA-4574 / FGSC 10173)	Cytoplasm
Pre-mRNA-splicing factor cwc22	Q7RX84	*Neurospora crassa* (strain ATCC 24698 / 74-OR23-1A / CBS 708.71 / DSM 1257 / FGSC 987)	Cytoplasm
Pre-mRNA-splicing ATP-dependent RNA helicase PRP28	Q1DMX8	*Coccidioides immitis* (strain RS)	Cytoplasm
Nucleolar protein 58	A2QE38	*Aspergillus niger* (strain ATCC MYA-4892 / CBS 513.88 / FGSC A1513)	Nucleolus
Nucleolar protein 9	A8P7F7	*Coprinopsis cinerea* (strain Okayama-7 / 130 / ATCC MYA-4618 / FGSC 9003)	Nucleolus

Putative magnesium-dependent phosphatase P8B7.31	O94279	*Schizosaccharomyces pombe* (strain 972 / ATCC 24843)	Cytosol
Mitochondrial group I intron splicing factor CCM1	C4Y2K4	*Clavispora lusitaniae* (strain ATCC 42720)	Mitochondria
Protein CFT1	Q6FSD2	*Candida glabrata* (strain ATCC 2001 / BCRC 20586 / JCM 3761 / NBRC 0622 / NRRL Y-65 / CBS 138)	Cytoplasm
Protein bfr2	Q7S6P8	*Neurospora crassa* (strain ATCC 24698 / 74-OR23-1A / CBS 708.71 / DSM 1257 / FGSC 987)	Nucleolus
Pre-mRNA-processing ATP-dependent RNA helicase PRP5	Q1DHB2	*Coccidioides immitis* (strain RS)	Nucleus
**Intracellular regulation**			
Rho-type GTPase-activating protein 1	P39083	*Saccharomyces cerevisiae* (strain ATCC 204508 / S288c)	Cytoplasm
AAA domain-containing protein	A0A2H3HEN3	*Fusarium oxysporum* f. sp. *radicis-cucumerinum*	Cytoplasm
Brr6_like_C_C domain-containing protein	A0A2H3H6T6	*Fusarium oxysporum* f. sp. *radicis-cucumerinum*	Nuclear Membrane
Serine/threonine-protein kinase ppk15	Q9P6P3	*Schizosaccharomyces pombe* (strain 972 / ATCC 24843)	Cytoplasm
26S proteasome regulatory subunit 4 homolog	P40327	*Saccharomyces cerevisiae* (strain ATCC 204508 / S288c)	Cytoplasm
Rho1 guanine nucleotide exchange factor TUS1	Q06412	*Saccharomyces cerevisiae* (strain ATCC 204508 / S288c)	Nucleus

Nucleolar GTP-binding protein 2	NOG2_CRYGA	*Cryptococcus gattii*	Nucleolus
Palmitoyltransferase AKR1	Q6C520	*Yarrowia lipolytica* (strain CLIB 122 / E 150)	Golgi membrane
**Transcription regulation**			
Transcription factor tau 55 kDa subunit	Q12415	*Saccharomyces cerevisiae* (strain ATCC 204508 / S288c)	Nucleus
Transcription initiation factor TFIID subunit 6	P53040	*Saccharomyces cerevisiae* (strain ATCC 204508 / S288c)	Cytosol
THO complex subunit HPR1	P17629	*Saccharomyces cerevisiae* (strain ATCC 204508 / S288c)	Chromosome
Zinc finger transcription factor YRM1	Q12340	*Saccharomyces cerevisiae* (strain ATCC 204508 / S288c)	Cytoplasm
Chromatin structure-remodeling complex subunit RSC9	Q03124	*Saccharomyces cerevisiae* (strain ATCC 204508 / S288c)	Chromatin
Regulatory protein SWI6	P09959	*Saccharomyces cerevisiae* (strain ATCC 204508 / S288c)	Cytoplasm
Transcriptional activator protein DAL81	P21657	*Saccharomyces cerevisiae* (strain ATCC 204508 / S288c)	Nucleus
Pyrimidine pathway regulatory protein 1	P07272	*Saccharomyces cerevisiae* (strain ATCC 204508 / S288c)	Nucleus
Transcriptional coactivator HFI1/ADA1	Q12060	*Saccharomyces cerevisiae* (strain ATCC 204508 / S288c)	Nucleus
Repression factor of MSEs protein 1	Q12192	*Saccharomyces cerevisiae* (strain ATCC 204508 / S288c)	Nucleus
26S proteasome regulatory subunit RPN14	P53196	*Saccharomyces cerevisiae* (strain ATCC 204508 / S288c)	Cytoplasm

Thiamine biosynthesis regulatory protein	P38141	*Saccharomyces cerevisiae* (strain ATCC 204508 / S288c)	Nucleus
Actin-related protein 7	Q12406	*Saccharomyces cerevisiae* (strain ATCC 204508 / S288c)	Chromatin
Histone deacetylase HOS3	Q02959	*Saccharomyces cerevisiae* (strain ATCC 204508 / S288c)	Cytoplasm
Maltose fermentation regulatory protein MAL13	P53338	*Saccharomyces cerevisiae* (strain ATCC 204508 / S288c)	Nucleus
CULLIN_2 domain-containing protein	A0A2H3GM29	*Fusarium oxysporum* f. sp. *radicis-cucumerinum*	Nucleus
Helo_like_N domain-containing protein	A0A2H3GL70	*Fusarium oxysporum* f. sp. *radicis-cucumerinum*	Unknown
Zn(2)-C6 fungal-type domain-containing protein	A0A2H3H1R0	*Fusarium oxysporum* f. sp. *radicis-cucumerinum*	Nucleus
ARID domain-containing protein	A0A2H3HSD9	*Fusarium oxysporum* f. sp. *radicis-cucumerinum*	Nucleus
BHLH domain-containing protein	A0A2H3H6H0	*Fusarium oxysporum* f. sp. *radicis-cucumerinum*	Unknown
Zn(2)-C6 fungal-type domain-containing protein	A0A2H3HMI4	*Fusarium oxysporum* f. sp. *radicis-cucumerinum*	Nucleus
HSF_DOMAIN domain-containing protein	A0A2H3H406	*Fusarium oxysporum* f. sp. *radicis-cucumerinum*	Nucleus
DNA-binding protein RAP1	A0A2H3HT92	*Fusarium oxysporum* f. sp. *radicis-cucumerinum*	Nucleus
Probable E3 ubiquitin ligase complex SCF subunit sconB	B6Q4Z5	*Talaromyces marneffei* (strain ATCC 18224 / CBS 334.59 / QM 7333)	Nucleus

Mediator of RNA polymerase II transcription subunit 12	Q751D2	*Ashbya gossypii* (strain ATCC 10895 / CBS 109.51 / FGSC 9923 / NRRL Y-1056)	Nucleus
Transcription regulatory protein SNF12	P53628	*Saccharomyces cerevisiae* (strain ATCC 204508 / S288c)	Nucleus
Multiprotein-bridging factor 1	Q6FJN0	*Candida glabrata* (strain ATCC 2001 / BCRC 20586 / JCM 3761 / NBRC 0622 / NRRL Y-65 / CBS 138)	Extracellular region
rDNA transcriptional regulator POL5	P39985	*Saccharomyces cerevisiae* (strain ATCC 204508 / S288c)	Nucleolus
RNA polymerase-associated protein LEO1	P38439	*Saccharomyces cerevisiae* (strain ATCC 204508 / S288c)	Nucleus
Sorbicillinoid biosynthetic cluster transcription factor sor4	G0R6T4	*Hypocrea jecorina* (strain QM6a)	Nucleus
Arabinanolytic transcriptional activator araR	A2QJX5	*Aspergillus niger* (strain ATCC MYA-4892 / CBS 513.88 / FGSC A1513)	Nucleus
Transcriptional regulatory protein SIN3	P22579	*Saccharomyces cerevisiae* (strain ATCC 204508 / S288c)	Nucleus
CCR4-Not complex 3'-5'-exoribonuclease subunit Ccr4	A1CIJ6	*Aspergillus clavatus* (strain ATCC 1007 / CBS 513.65 / DSM 816 / NCTC 3887 / NRRL 1 / QM 1276 / 107)	Cytoplasm
Transcription factor kpeA	Q2UJJ4	*Aspergillus oryzae* (strain ATCC 42149 / RIB 40)	Nucleus
Mating-type locus allele B3 protein	P22017	*Ustilago maydis*	Nucleus
Putative transcriptional activator MSA2	P36157	*Saccharomyces cerevisiae* (strain ATCC 204508 / S288c)	Cytoplasm

SWI/SNF global transcription activator complex subunit SWP82	P43554	*Saccharomyces cerevisiae* (strain ATCC 204508 / S288c)	Chromatin
DNA-binding protein creA	Q01981	*Emericella nidulans* (strain FGSC A4 / ATCC 38163 / CBS 112.46 / NRRL 194 / M139)	Cytoplasm
General transcription and DNA repair factor IIH subunit tcf-29	Q9P5N7	*Neurospora crassa* (strain ATCC 24698 / 74-OR23-1A / CBS 708.71 / DSM 1257 / FGSC 987)	Cytoplasm
Mating-type protein A-alpha Y3	P37934	*Schizophyllum commune*	Nucleus
**DNA repair**			
Flap endonuclease 1	P26793	*Saccharomyces cerevisiae* (strain ATCC 204508 / S288c)	Cytoplasm
Bromodomain-containing factor 2	Q07442	*Saccharomyces cerevisiae* (strain ATCC 204508 / S288c)	Nucleus
E3 SUMO-protein ligase MMS21	P38632	*Saccharomyces cerevisiae* (strain ATCC 204508 / S288c)	Chromosome
Exodeoxyribonuclease 1	P39875	*Saccharomyces cerevisiae* (strain ATCC 204508 / S288c)	Cytoplasm
Probable DNA-binding protein SNT1	P25357	*Saccharomyces cerevisiae* (strain ATCC 204508 / S288c)	Cytosol
Deoxyribodipyrimidine photo-lyase, mitochondrial	P05066	*Saccharomyces cerevisiae* (strain ATCC 204508 / S288c)	Cytoplasm
DNA ligase	A0A2H3GHI8	*Fusarium oxysporum* f. sp. *radicis-cucumerinum*	Nucleus
DNA polymerase lambda	A0A2H3I9G2	*Fusarium oxysporum* f. sp. *radicis-cucumerinum*	Nucleus
Structure-specific endonuclease subunit SLX4	A0A2H3HWW5	*Fusarium oxysporum* f. sp. *radicis-cucumerinum*	Nucleus

RNase H type-1 domain-containing protein	A0A2H3FPM5	*Fusarium oxysporum* f. sp. *radicis-cucumerinum*	Nucleus
Proliferating cell nuclear antigen	P15873	*Saccharomyces cerevisiae* (strain ATCC 204508 / S288c)	Nucleus
Serine/threonine-protein kinase MEC1	Q59LR2	*Candida albicans* (strain SC5314 / ATCC MYA-2876)	Nucleus
General transcription and DNA repair factor IIH subunit tfb1	O94684	*Schizosaccharomyces pombe* (strain 972 / ATCC 24843)	Nucleus
PH domain-containing protein YHR131C	P38838	*Saccharomyces cerevisiae* (strain ATCC 204508 / S288c)	Nucleus
DNA topoisomerase 2	P08096	*Schizosaccharomyces pombe* (strain 972 / ATCC 24843)	Nucleus
**Intracellular Protein Transport**			
Conserved oligomeric Golgi complex subunit 4	Q06096	*Saccharomyces cerevisiae* (strain ATCC 204508 / S288c)	Cytosol
Phosphatidylinositol 4-kinase LSB6	P42951	*Saccharomyces cerevisiae* (strain ATCC 204508 / S288c)	Cytoplasm
Tubulin-specific chaperone C	P46670	*Saccharomyces cerevisiae* (strain ATCC 204508 / S288c)	Cytoplasm
Pheromone-processing carboxypeptidase KEX1	P09620	*Saccharomyces cerevisiae* (strain ATCC 204508 / S288c)	Membrane
Rho1 guanine nucleotide exchange factor TUS1	Q06412	*Saccharomyces cerevisiae* (strain ATCC 204508 / S288c)	Membrane
Vacuolar protein-sorting-associated protein 24	P36095	*Saccharomyces cerevisiae* (strain ATCC 204508 / S288c)	Cytoplasm
Protein MLP2	P40457	*Saccharomyces cerevisiae* (strain ATCC 204508 / S288c)	Mitochondria

Nucleoporin NUP170	P38181	*Saccharomyces cerevisiae* (strain ATCC 204508 / S288c)	Nuclear membrane
Importin subunit beta-5	P53067	*Saccharomyces cerevisiae* (strain ATCC 204508 / S288c)	Cytoplasm
Mitochondrial inner membrane protease subunit 1	P28627	*Saccharomyces cerevisiae* (strain ATCC 204508 / S288c)	Mitochondrial Inner Membrane
Protein OS-9 homolog	Q99220	*Saccharomyces cerevisiae* (strain ATCC 204508 / S288c)	Endoplasmic reticulum
Midasin	Q12019	*Saccharomyces cerevisiae* (strain ATCC 204508 / S288c)	Mitochondria
Dynein heavy chain, cytoplasmic	P36022	*Saccharomyces cerevisiae* (strain ATCC 204508 / S288c)	Cytoplasm
Serine/threonine-protein kinase VPS15	P22219	*Saccharomyces cerevisiae* (strain ATCC 204508 / S288c)	Membrane
Protein LST4	Q757Y7	*Ashbya gossypii* (strain ATCC 10895 / CBS 109.51 / FGSC 9923 / NRRL Y-1056)	Vacuolar membrane
AP-3 complex subunit mu	P38153	*Saccharomyces cerevisiae* (strain ATCC 204508 / S288c)	Membrane
Protein transport protein sec72	Q9P7V5	*Schizosaccharomyces pombe* (strain 972 / ATCC 24843)	Cytoplasm
Vacuolar protein sorting/targeting protein 10	B9W9N8	*Candida dubliniensis* (strain CD36 / ATCC MYA-646 / CBS 7987 / NCPF 3949 / NRRL Y-17841)	Membrane
Vacuolar membrane protein pep3	O74925	*Schizosaccharomyces pombe* (strain 972 / ATCC 24843)	Cytoplasm
Probable metabolite transport protein YFL040W	P43562	*Saccharomyces cerevisiae* (strain ATCC 204508 / S288c)	Membrane
Myosin-1	Q7Z8J6	*Ustilago maydis* (strain 521 / FGSC 9021)	Cytoplasm

Clustered mitochondria protein homolog	A5DWP3	*Lodderomyces elongisporus* (strain ATCC 11503 / CBS 2605 / JCM 1781 / NBRC 1676 / NRRL YB-4239)	Cytoplasm
Regulator of V-ATPase in vacuolar membrane protein 2	Q03956	*Saccharomyces cerevisiae* (strain ATCC 204508 / S288c)	Endossome
Golgi SNAP receptor complex member 1	P38736	*Saccharomyces cerevisiae* (strain ATCC 204508 / S288c)	Golgi membrane
Probable nucleolar GTP-binding protein 1	O94659	*Schizosaccharomyces pombe* (strain 972 / ATCC 24843)	Nucleolus
ERAD-associated E3 ubiquitin-protein ligase component HRD3	Q05787	*Saccharomyces cerevisiae* (strain ATCC 204508 / S288c)	Endoplasmic reticulum
**Lipid metabolism**			
Sterol 3-beta-glucosyltransferase	Q06321	*Saccharomyces cerevisiae* (strain ATCC 204508 / S288c)	Cytoplasm
Acyl-coenzyme A diphosphatase SCS3	P53012	*Saccharomyces cerevisiae* (strain ATCC 204508 / S288c)	Endoplasmic reticulum
2-acyl-1-lysophosphatidylinositol acyltransferase	P38226	*Saccharomyces cerevisiae* (strain ATCC 204508 / S288c)	Endoplasmic reticulum
Dihydrosphingosine 1-phosphate phosphatase YSR3	P23501	*Saccharomyces cerevisiae* (strain ATCC 204508 / S288c)	Endoplasmic reticulum
NADPH--cytochrome P450 reductase	A0A2H3GQQ7	*Fusarium oxysporum* f. sp. *radicis-cucumerinum*	Endoplasmic reticulum
Highly reducing polyketide synthase ntnH	A0A455M2Y3	*Nectria* sp.	Cytoplasm

Fusarin C synthetase	S0EEY3	*Gibberella fujikuroi* (strain CBS 195.34 / IMI 58289 / NRRL A-6831)	Cytoplasm
Phosphatidylethanolamine N-methyltransferase	D1ZIW5	*Sordaria macrospora* (strain ATCC MYA-333 / DSM 997 / K(L3346) / K-hell)	Endoplasmic reticulum
Non-reducing polyketide synthase atr1	A0A8F4SKJ7	*Stereocaulon alpinum*	Cytoplasm
Very-long-chain 3-oxoacyl-CoA reductase	Q6FRM0	*Candida glabrata* (strain ATCC 2001 / BCRC 20586 / JCM 3761 / NBRC 0622 / NRRL Y-65 / CBS 138)	Membrane endoplasmic reticulum
Hybrid PKS-NRPS synthetase phm1	A0A2Z5XAL7	*Pyrenochaetopsis sp.*	Unknown
Highly reducing polyketide synthase easB	Q5BA83	*Emericella nidulans* (strain FGSC A4 / ATCC 38163 / CBS 112.46 / NRRL 194 / M139)	Unknown
Phosphatidylserine decarboxylase proenzyme 3	O14111	*Schizosaccharomyces pombe* (strain 972 / ATCC 24843)	Cytosol
Phosphatidylinositol 4-kinase STT4	P37297	*Saccharomyces cerevisiae* (strain ATCC 204508 / S288c)	Cytoplasm
**Protein biosynthesis**			
Translation machinery-associated protein 22	P47089	*Saccharomyces cerevisiae* (strain ATCC 204508 / S288c)	Cytoplasm
37S ribosomal protein S7, mitochondrial	P47150	*Saccharomyces cerevisiae* (strain ATCC 204508 / S288c)	Mitochondrial Inner Membrane
E3 ubiquitin-protein ligase UBR1	P19812	*Saccharomyces cerevisiae* (strain ATCC 204508 / S288c)	Cytoplasm
D-aminoacyl-tRNA deacylase	A0A2H3HCC5	*Fusarium oxysporum* f. sp. *radicis-cucumerinum*	Cytoplasm

Deoxyhypusine hydroxylase	A0A2H3I3Q0	*Fusarium oxysporum* f. sp. *radicis-cucumerinum*	Cytoplasm
Large ribosomal subunit protein eL8A	A0A1D8PF11	*Candida albicans* (strain SC5314 / ATCC MYA-2876)	Cytoplasm
Lon protease homolog 2, peroxisomal	Q6CWS4	*Kluyveromyces lactis* (strain ATCC 8585 / CBS 2359 / DSM 70799 / NBRC 1267 / NRRL Y-1140 / WM37)	Endossome
Eukaryotic translation initiation factor 3 subunit A	Q6CT90	*Kluyveromyces lactis* (strain ATCC 8585 / CBS 2359 / DSM 70799 / NBRC 1267 / NRRL Y-1140 / WM37)	Cytoplasm
Presequence protease, mitochondrial	Q6BTC0	*Debaryomyces hansenii* (strain ATCC 36239 / CBS 767 / BCRC 21394 / JCM 1990 / NBRC 0083 / IGC 2968)	Mitochondrial Inner Membrane
Mitochondrial intermediate peptidase	Q2UN31	*Aspergillus oryzae* (strain ATCC 42149 / RIB 40)	Mitochondrial matrix
Probable Xaa-Pro aminopeptidase P	A7E4T8	*Sclerotinia sclerotiorum* (strain ATCC 18683 / 1980 / Ss-1)	Cytoplasm
**Nucleotide metabolism**			
Adenylosuccinate synthetase	P80210	*Saccharomyces cerevisiae* (strain ATCC 204508 / S288c)	Cytoplasm
S-methyl-5'-thioadenosine phosphorylase	Q07938	*Saccharomyces cerevisiae* (strain ATCC 204508 / S288c)	Cytoplasm
GFO_IDH_MocA domain-containing protein	A0A2H3H014	*Fusarium oxysporum* f. sp. *radicis-cucumerinum*	Cytoplasm
Thymidylate kinase	P36590	*Schizosaccharomyces pombe* (strain 972 / ATCC 24843)	Cytoplasm
**Cell wall organization**			
Plasma membrane ATPase proteolipid 2	P40169	*Saccharomyces cerevisiae* (strain ATCC 204508 / S288c)	Cellular Periphery

1,3-beta-glucanosyltransferase gel4	B0XVI5	*Aspergillus fumigatus* (strain CBS 144.89 / FGSC A1163 / CEA10)	Plasm Membrane
Beta-mannosyltransferase 8	Q5AHD6	*Candida albicans* (strain SC5314 / ATCC MYA-2876)	Membrane
Serine/threonine-protein kinase ksg1	Q12701	*Schizosaccharomyces pombe* (strain 972 / ATCC 24843)	Cytoplasm
**Metabolism and energy**			
Ferulic acid decarboxylase 1	Q03034	*Saccharomyces cerevisiae* (strain ATCC 204508 / S288c)	Cytoplasm
Thiosulfate sulfurtransferase RDL2, mitochondrial	Q08742	*Saccharomyces cerevisiae* (strain ATCC 204508 / S288c)	Mitochondria
Iron-sulfur assembly protein 1	Q07821	*Saccharomyces cerevisiae* (strain ATCC 204508 / S288c)	Cytoplasm
Isocitrate lyase	P20014	*Candida tropicalis*	Glyoxysome
Acetamidase	Q12559	*Aspergillus oryzae* (strain ATCC 42149 / RIB 40)	Cytoplasm
Probable cytosolic iron-sulfur protein assembly protein 1	Q5BDJ5	*Emericella nidulans* (strain FGSC A4 / ATCC 38163 / CBS 112.46 / NRRL 194 / M139)	Cytosol
Trans-aconitate decarboxylase 1	A0A0U2UYC4	*Ustilago maydis*	Cytosol
**Carbohydrate metabolism**			
Putative endoplasmic reticulum mannosidase MNL2	Q12205	*Saccharomyces cerevisiae* (strain ATCC 204508 / S288c)	Endoplasmic reticulum
Inositol hexakisphosphate and diphosphoinositol-pentakisphosphate kinase	Q06685	*Saccharomyces cerevisiae* (strain ATCC 204508 / S288c)	Cytoplasm

Glyco_hydro_32N domain-containing protein	A0A2H3HH14	*Fusarium oxysporum* f. sp. *radicis-cucumerinum*	Cytoplasm
Probable alpha/beta-glucosidase agdC	A1CNK4	*Aspergillus clavatus* (strain ATCC 1007 / CBS 513.65 / DSM 816 / NCTC 3887 / NRRL 1 / QM 1276 / 107)	Extracellular region
**Oxireductase**			
Amino_oxidase domain-containing protein	A0A2H3GTT9	*Fusarium oxysporum* f. sp. *radicis-cucumerinum*	Unknown
Aldedh domain-containing protein	A0A2H3H922	*Fusarium oxysporum* f. sp. *radicis-cucumerinum*	Unknown
DSBA domain-containing protein	A0A2H3GX46	*Fusarium oxysporum* f. sp. *radicis-cucumerinum*	Unknown
Dehydrogenase efuE	A0A2Z4HPZ6	*Hormonema carpetanum*	Unknown
FAD-linked oxidoreductase fogF	A0A017SGC7	*Aspergillus ruber* (strain CBS 135680)	Unknown
Dehydrogenase GME11362	A0A5B8YW44	*Pestalotiopsis microspora*	Unknown
NADH-cytochrome b5 reductase 1	A6R2K7	*Ajellomyces capsulatus* (strain NAm1 / WU24)	Mitochondrial outer membrane
Hybrid PKS-NRPS synthetase poxE	S7ZFK6	*Penicillium oxalicum* (strain 114-2 / CGMCC 5302)	Unknown
**Metabolism and synthesis of amino acids**			
D-3-phosphoglycerate dehydrogenase	A0A2H3HLY6	*Fusarium oxysporum* f. sp. *radicis-cucumerinum*	Unknown
Dihydrolipoyllysine-residue succinyltransferase	A0A2H3H0E4	*Fusarium oxysporum* f. sp. *radicis-cucumerinum*	Unknown

Peptidase S1 domain-containing protein	A0A2H3G9J0	*Fusarium oxysporum* f. sp. *radicis-cucumerinum*	Cytoplasm
Pyr_redox_2 domain-containing protein	A0A2H3GL97	*Fusarium oxysporum* f. sp. *radicis-cucumerinum*	Cytoplasm
HpcH_HpaI domain-containing protein	A0A2H3HID5	*Fusarium oxysporum* f. sp. *radicis-cucumerinum*	Cytoplasm
3-isopropylmalate dehydrogenase B	P87257	*Aspergillus niger*	Cytoplasm
FAD-dependent monooxygenase nscC	E4V2N4	*Arthroderma gypseum* (strain ATCC MYA-4604 / CBS 118893)	Cytoplasm
Trimethyllysine dioxygenase	Q96UB1	*Neurospora crassa* (strain ATCC 24698 / 74-OR23-1A / CBS 708.71 / DSM 1257 / FGSC 987)	Mitochondria
Thiamine pyrophosphokinase	P41888	*Schizosaccharomyces pombe* (strain 972 / ATCC 24843)	Cytoplasm
**Virulence**			
Subtilisin-like protease 5	C5G1D1	*Arthroderma otae* (strain ATCC MYA-4605 / CBS 113480)	Extracellular region
Tryprostatin B synthase	B9WZX3	*Aspergillus fumigatus*	Extracellular region
Short-chain dehydrogenase/reductase phmF	Q0V6Q2	*Phaeosphaeria nodorum* (strain SN15 / ATCC MYA-4574 / FGSC 10173)	Unknown
**Gene regulation**			
ISWI chromatin-remodeling complex ATPase ISW2	Q5A310	*Candida albicans* (strain SC5314 / ATCC MYA-2876)	Nucleus

ATPase synthesis protein 25, mitochondrial	Q6C7D6	*Yarrowia lipolytica* (strain CLIB 122 / E 150)	Mitochondrial Inner Membrane
**Secondary Metabolism**			
Tenellin synthetase	J4KMC1	*Beauveria bassiana* (strain ARSEF 2860)	Cytoplasm
Nonribosomal peptide synthase agiA	B8NY88	*Aspergillus flavus* (strain ATCC 200026 / FGSC A1120 / IAM 13836 / NRRL 3357 / JCM 12722 / SRRC 167)	Cytoplasm
Highly reducing polyketide synthase tazB	Q0CS88	*Aspergillus terreus* (strain NIH 2624 / FGSC A1156)	Cytoplasm
N-(5-amino-5-carboxypentanoyl)-L-cysteinyl-D-valine synthase	P25464	*Acremonium chrysogenum*	Cytosol
Hispidin synthase	A0A3G9K3K9	*Neonothopanus nambi*	Cytoplasm
Acyl-CoA ligase cnsG	A0A0A2J5U8	*Penicillium expansum*	Unknown
Nonribosomal peptide synthase atnA	Q5AUZ6	*Emericella nidulans* (strain FGSC A4 / ATCC 38163 / CBS 112.46 / NRRL 194 / M139)	Cytoplasm
Nonribisomal peptide synthetase benY	P9WEU8	*Aspergillus terreus*	Unknown
**Unknown**			
HET domain-containing protein	A0A2H3H351	*Fusarium oxysporum* f. sp. *radicis-cucumerinum*	Unknown
SCP domain-containing protein	A0A2H3FUN6	*Fusarium oxysporum* f. sp. *radicis-cucumerinum*	Unknown
UDENN domain-containing protein	A0A2H3HHF6	*Fusarium oxysporum* f. sp. *radicis-cucumerinum*	Unknown

PS_pyruv_trans domain-containing protein	A0A2H3GYW2	*Fusarium oxysporum* f. sp. *radicis-cucumerinum*	Unknown
DRIM domain-containing protein	A0A2H3HTW8	*Fusarium oxysporum* f. sp. *radicis-cucumerinum*	Unknown
HET domain-containing protein	A0A2H3I5C6	*Fusarium oxysporum* f. sp. *radicis-cucumerinum*	Unknown
DJ-1_PfpI domain-containing protein	A0A2H3GJB5	*Fusarium oxysporum* f. sp. *radicis-cucumerinum*	Unknown
CHAT domain-containing protein	A0A2H3H2S4	*Fusarium oxysporum* f. sp. *radicis-cucumerinum*	Unknown
C2H2-type domain-containing protein	A0A2H3FUZ0	*Fusarium oxysporum* f. sp. *radicis-cucumerinum*	Unknown
BTB domain-containing protein	A0A2H3GTC7	*Fusarium oxysporum* f. sp. *radicis-cucumerinum*	Unknown
CIA30 domain-containing protein	A0A2H3GUN4	*Fusarium oxysporum* f. sp. *radicis-cucumerinum*	Unknown
2EXR domain-containing protein	A0A2H3GDT7	*Fusarium oxysporum* f. sp. *radicis-cucumerinum*	Unknown
CHK domain-containing protein	A0A2H3HAB8	*Fusarium oxysporum* f. sp. *radicis-cucumerinum*	Unknown
zf-Mss51 domain-containing protein	A0A2H3H634	*Fusarium oxysporum* f. sp. *radicis-cucumerinum*	Unknown
PALP domain-containing protein	A0A2H3GJ03	*Fusarium oxysporum* f. sp. *radicis-cucumerinum*	Unknown
AB hydrolase-1 domain-containing protein	A0A2H3I1B9	*Fusarium oxysporum* f. sp. *radicis-cucumerinum*	Unknown
Altered inheritance of mitochondria protein 24, mitochondrial	B3LQG6	*Saccharomyces cerevisiae* (strain RM11-1a)	Mitochondria
Protein yippee-like MOH1	P38191	*Saccharomyces cerevisiae* (strain ATCC 204508 / S288c)	Unknown
